# The Role of Risk Factors for the Progression of Patients with T1b-T2 Papillary Thyroid Carcinoma (PC) during Long-Term Follow-Up

**DOI:** 10.3390/jcm13185373

**Published:** 2024-09-11

**Authors:** Andrea Marongiu, Susanna Nuvoli, Andrea De Vito, Antonio Mura, Sonia Vargiu, Angela Spanu, Giuseppe Madeddu

**Affiliations:** 1 Unit of Nuclear Medicine, Department of Medicine, Surgery and Pharmacy, University of Sassari, 07100 Sassari, Italy; amarongiu2@uniss.it (A.M.); a.mura203@studenti.uniss.it (A.M.); sovargiu@tiscali.it (S.V.); aspanu@uniss.it (A.S.); giuseppe.madeddu@email.it (G.M.); 2 Unit of Infectious Diseases, Department of Medicine, Surgery and Pharmacy, University of Sassari, 07100 Sassari, Italy; andreadevitoaho@gmail.com

**Keywords:** T1b-T2 papillary thyroid carcinoma (PC), *AJCC*/TNM classification, risk factors, minimal extrathyroid tumor extension (mETE), neck lymph node (LN) metastasis, multifocality/multicentricity (M/M), recurrence, disease-free survival (DFS), total thyroidectomy, ^131^I-SPECT/CT

## Abstract

**Background/Objectives**: Recurrence prediction for patients with PC and tumor sizes ranging between 1 and 4 cm, classified as T1b and T2, remains a controversial problem. We evaluated which risk factors, identified during the primary tumor surgery, might play a prognostic role in predicting disease progression. **Methods**: We retrospectively enrolled 363 patients with classic PC who were in follow-up (207 T1b, 156 T2), with tissue risk factors at surgery in 209/363 cases. In all cases, an ^131^I-whole-body scan, SPECT/CT, and US were employed to detect any metastases during follow-up, and histology was used to confirm lesions. In the absence of surgery, metastases were validated by radioisotopic and radiologic procedures, eventually culminating in a needle biopsy and sequential thyroglobulin changes. **Results**: Metastases occurred in 61/363 (16.8%) patients (24 T1b, 37 T2). In 50/61 cases, the following risk factors were identified: minimal extrathyroid tumor extension (mETE) alone in 12/50 patients, neck lymph node (LN) metastases in 8/50 cases, and multifocality/multicentricity (M/M) in 6/50 cases. In the remaining 24/50 cases, the risk factors were associated with each other. From a Cox regression multivariate analysis, metastasis development was significantly (*p* < 0.001) influenced by only mETE and LN metastases, with a shorter disease-free survival (log-rank test). **Conclusions**: The current study proves that mETE and neck LN metastases are associated with aggressive PC. While LN metastasis’ role is known, mETE’s role is still being debated, and was removed by the *AJCC*’s eighth edition because it was considered to not be associated with an unfavorable prognosis. However, this interpretation is not supported by the present study and, according to comparable studies, we suggest a revision of the mETE classification be considered in the next *AJCC* edition.

## 1. Introduction

Papillary thyroid cancer (PC) is the most common form of thyroid cancer [1,2], and its incidence has increased in recent years [3,4,5,6,7,8]; it is generally characterized by a slow growth rate, a favorable outcome, and a very low cancer-specific mortality [9,10,11,12,13]. However, PC can also have a poorer prognosis in some cases, depending on the presence of risk factors identified during the primary tumor surgery that can predict recurrences and metastases during follow-up. Among these risk factors, the presence of neck lymph node (LN) metastases at diagnosis is the risk factor with the most elevated impact on the appearance of metastases during the disease and on shorter disease-free survival (DFS) [13,14,15,16,17,18,19]. Moreover, it is well known that extrathyroid tumor extension (ETE) and, in particular, extended ETE (eETE), which is characterized by tumor invasion of the subcutaneous soft tissue, larynx, recurrent laryngeal nerve, trachea, and esophagus, is considered to be responsible for unfavorable PC prognoses [20,21,22] and, in particular, in cases for those aged over 50 years old [23]. This risk factor can be retained as an independent predictor of shortened DFS, and as also having a relationship with mortality in some cases. This risk factor, according to the American Thyroid Association (ATA) Management Guidelines [24], is associated with a high risk of recurrence and metastases. However, minimal ETE (mETE), whose characteristics are represented by tumor extension beyond the thyroid capsule, perithyroid soft tissue, and sternothyroid muscle invasion, and that can be detected microscopically through histology, is associated with an intermediate risk, and its role is still under discussion due to controversial results. In the seventh *AJCC*, mETE was recognized as a significant risk factor and designated as T3. Still, in the eighth *AJCC*, the T3 designation was removed due to the assumption that it was not connected to an unfavorable tumor prognosis. Thus, mETE in the *AJCC* eighth edition was not considered in either the T-category or the stadium definition [25].

In the present study, in a group of T1b-T2 patients with a tumor size larger than 10 mm and equal to or lower than 40 mm, classified according to the *AJCC* eighth edition, and who have undergone a total thyroidectomy and radioiodine ablation, the prognostic role of some risk factors identified during surgery was examined. We evaluated, particularly, whether neck LN metastases, mETE, and multifocality/multicentricity (M/M) may be predictive factors for metastases during follow-up, with an impact on DFS. The prognostic role of tumor size, age, and gender were also considered in this group of patients.

## 2. Materials and Methods

### 2.1. Patients

As shown in Table 1, during the period of time between 2006 and 2021, 363 consecutive adult patients with classic T1b-T2 PC variants, with a size larger than 10 mm and equal to or lower than 40 mm, were enrolled in a retrospective study with a follow-up period of approximately 8–10 years after a total thyroidectomy and radioiodine ablation.

As illustrated in Table 2, one or more risk factors were present during surgery in 209/363 (57.6%) patients with PC (114 T1b, 95 T2).

No risk factors were ascertained in the remaining 154/363 patients with PC (93 T1b and 61 T2).

The exclusion criteria at the time of the surgery on the primary tumor included a papillary microcarcinoma (PTMC) with a diameter ≤10 mm, a PC larger than 40 mm in diameter, aggressive PC variants, eETE, and distant metastases. The patients with a follow-up <12 months from therapy were excluded.

Surgery was performed in the 282/363 patients with thyroid nodules because of the high suspicion of DTC at the fine-needle aspiration biopsy (FNAB), afterwards ascertained by histology, but in the remaining 81/363 patients who submitted to surgery for a multinodular goiter, the carcinomas were identified only at histology.

AbTg, AbTPO, and TRAbs in the serum, as well as histological abnormalities due to thyroid autoimmune diseases associated with PC, did not show up in any of the patients. Additionally, all the patients were in clinically euthyroid status, with normal thyroid function tests that excluded hyperthyroidism and hypothyroidism conditions; thus, there was no specific pharmacological therapy administered before surgery.

The therapeutic strategy chosen by the University Surgery Department resulted in total thyroidectomies, all performed in the same Surgical Unit. No patients experienced postoperative complications, like hemorrhages, laryngeal nerve invasion, dysphagia, diplegia, hypocalcemia, infection, etc., with stable disorders. All the patients received radioiodine ablation after surgery.

In 79 patients (30 T1b group and 49 T2 group), the LN was taken out of the central compartment of the neck (N1a). In 18 cases (7 T1b group and 11 T2 group), it was taken out of the lateral regions of the neck (N1b), and both the central and lateral regions (N1a-N1b) were taken out in 38 cases (18 T1b group and 20 T2 group).

The LNs were removed only if they were suspected of being cancerous from imaging procedures before or during surgery. In this regard, therapeutic but not prophylactic lymph node dissection was performed in all these patients.

LN metastases were found in 58 patients (28 T1b and 30 T2). The central lymph node compartment was the primary site of metastasis in 12/58 patients (N1a), the lateral region in 25/58 cases (N1b), and both the central and lateral compartments in 21/58 patients (N1a-N1b).

The number of metastatic lymph nodes varied from a minimum of 1 to a maximum of 28 in individual patients, with a median of 3 (IQR 1–7).

There were no instances of extra-nodal extension; only infiltration of the lymph node capsule was reported in a few patients.

### 2.2. Methods

In all the patients, a clinical investigation, neck ultrasound (US), ^131^I-Whole-Body Scan (WBS) with a speed of 5 cm/min, and single-photon emission computerized tomography/computerized tomography (SPECT/CT over 360°, 180 per head, 3° angular step, matrix of 128 × 128, acquisition time of 40 s/frame, 1–1.2 zoom factor) were employed to detect eventual metastases during the follow-up. Following an injection of 185 MBq radioiodine by a hybrid dual-head gamma-ray system, nuclear medicine procedures were carried out within 24–48 h, and 72 h if necessary. The patients were in hypothyroidism after withdrawing L-thyroxine in 150 cases, or submitted to recombinant thyroid stimulating hormone (rh-TSH) in 213 cases. The TSH serum levels before the ^131^I scintigraphy were always over 50 µU/mL. Complementary exams, such as CT, MRI, and ^18^F-FDG PET/CT, were carried out on certain patients, if necessary.

The chemiluminescent immunoassay method was used to measure the serum TSH, thyroglobulin, and AbTg levels. The maximum amount of thyroglobulin that could be detected was 0.1 ng/mL. During the suppressive therapy, the thyroglobulin limit was considered to be <0.2 ng/mL, and after TSH stimulation, it was considered to be <1 ng/mL. The limit for the AbTg cutoff was 100 IU/mL.

All the radioisotopic instrumental examinations during follow-up were conducted at the Nuclear Medicine Center at the University Hospital where the current study was performed. Physicians A.M., S.N., A.S., and G.M. knew the reasons for the exams, but they were not aware of the results of previous investigations. The resolution of disagreements was achieved through consensus and there was minimal variability between the observers. The other imaging exams were carried out by the Radiologic Center, while in “vitro” tests were performed by the Central Endocrinological Laboratory of the same University Hospital.

### 2.3. Statistics

The Shapiro–Wilk test was applied to evaluate the continuous data distribution for normality. The continuous variables were presented using the means and standard deviations (SD), or the medians with interquartile ranges (IQRs, 25–75%). For the categorical data, frequencies as absolute numbers and percentages were expressed. The Mann–Whitney U test or Student’s *t*-test was used to compare the continuous variables across the different subgroups as appropriate. The differences in the categorical variables were assessed using Pearson’s chi-square test or Fisher’s exact test when required.

Further, a Cox proportional hazards regression analysis was conducted to explore the association between the various predictors and the risk of metastasis, treating metastasis as the dependent outcome.

For the graphical representation of the 10-year disease-free survival rates, Kaplan–Meier survival curves were utilized, with the log-rank test determining the statistical differences between the patient groups with and without significant risk factors. Results were considered to be statistically significant at a *p*-value of less than 0.05. All the statistical analyses were performed using STATA software, version 16.1 (StataCorp. LLC, College Station, TX, USA).

## 3. Results

The primary tumor was surgically analyzed to evaluate the relationship between mETE, neck LN metastases, M/M, the age of patients, and tumor size before considering the patient follow-up.

The global recognition of mETE involved 106/363 patients; 58/363 had neck LN metastases and 117/363 had M/M.

Regarding age, the 106 patients with mETE were older than the 257 without mETE, but the difference was not significant [52.5 (IQR 41–59) vs. 48 (IQR 38–58) years; *p* = 0.08]. However, the 58 patients with neck LN metastasis showed a significantly lower age compared to the 305 patients without LN metastasis [44 (IQR 28–53) vs. 49 (IQR (40–61) years; *p* < 0.001], while the difference was not statistically significant [48 (IQR 38–58) vs. 49.5 (IQR 39–59) years; *p* = 0.379] between the 117 patients with M/M and the 246 patients without M/M.

The patients with mETE had tumors that were significantly larger compared to the patients without mETE [21 (IQR 15–25) vs. 15 (IQR 12–25) mm; *p* = 0.008], but there was no significant difference found among the patients with LN metastasis [21.5 (IQR 15–25) vs. 17 (IQR 12–25) mm; *p* = 0.09] and M/M [16 (IQR 12–25) vs. 19.5 (IQR 13–25) mm; *p* = 0.451].

After thyroidectomy and subsequent radioiodine ablation, all the patients were subjected to a long-term follow-up, with a median of 100 (IQR 58–125) months. The follow-up did not result in the loss of any patients.

During the follow-up, metastases were detected in 61/363 (16.8%) patients. Neoplastic lesions were identified at least 18 months after the radioiodine ablation, to avoid these lesions being classified as a persistent disease if they were identified earlier, and were confirmed by histology. In case the latter exam was unavailable, the confirmation was made through the diagnostic imaging procedures mentioned above. Among these, an ^131^I SPECT/CT was also included after an eventual second therapeutic dose, an US with FNAB, when the foci were easily accessible. A sequential evaluation of serum thyroglobulin changes was performed at intervals of 6–9 months in the first two years after surgery, and annually subsequently, and whenever suspect signs of metastases occurred.

Among the 61 patients who experienced metastases, 24/61 (39.3%) were in the T1b group and 37/61 (60.7%) were in the T2 group, 23/61 (37.7%) were male and 38/61 (62.3%) were female, and 43/61 (70.5%) were aged under 55 years and 18/61 (29.5%) were aged over 55 years. There were 14/61 (23%) patients affected by goiter and 47/61 (77%) by single nodules. The SPECT/CT identified 110 metastases in the 61 patients, including 31 lesions in 24 T1b cases and 79 lesions in 37 T2 cases. The majority of the metastases (89/110) were located in the neck LN (29 in T1b patients and 60 in T2 patients); of these, 45 LN metastases were laterocervical (LTC), 16 sub/retromandibular (SM), 19 paratracheal (PT), and 9 supraclavicular (SC). The other 21/110 were distant metastases (9 lung, 6 mediastinum, 2 rib, 2 spine, and 2 pelvic muscle). A WBS visualized 24/89 neck LN metastases and 7/21 distant metastases (4 lung, 1 mediastinum, and 2 pelvic muscles); all of these were also detected by SPECT/CT, with statistically significant differences between the two procedures (*p* < 0.001). Moreover, a neck US identified neoplastic lymph nodes in 90.2% of the 61 patients, was in doubt in 6.5% of the cases, and presented reactive features in 3.3% of the cases. Furthermore, distant metastases developed in 11/61 patients and lesions were more frequently diagnosed in T2 patients (18 lesions in 8 patients) than in T1b (1 lesion each in 3 patients), with a median follow-up of 120 months.

**Figure 1:**
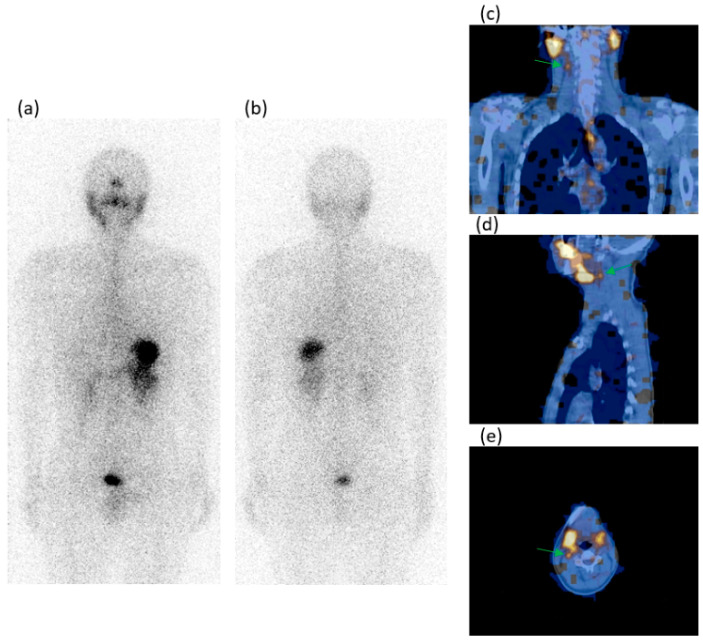
A 28-year-old male patient who underwent a total thyroidectomy and radioiodine ablation for a 15 mm T1b papillary carcinoma (PC) classic variant. At surgery, mETE and neck LN laterocervical metastasis were identified. During the follow-up, the WBS in anterior/posterior (**a**,**b**) views did not detect any foci of pathologic radioiodine uptake. The SPECT/CT on the coronal/sagittal/transaxial (**c**–**e**) slides evidenced a radioiodine avid focus (green arrows) in the neck (right laterocervical region), which on the sagittal slides was located behind the submandibular salivary gland. This focus was classified as a metastatic lymph node and was treated with a second surgery and radioiodine ablation. Thyroglobulin levels were in hypothyroidism range before the present scintigraphy: 6.6 ng/mL; AbTg: absent.

**Figure 2:**
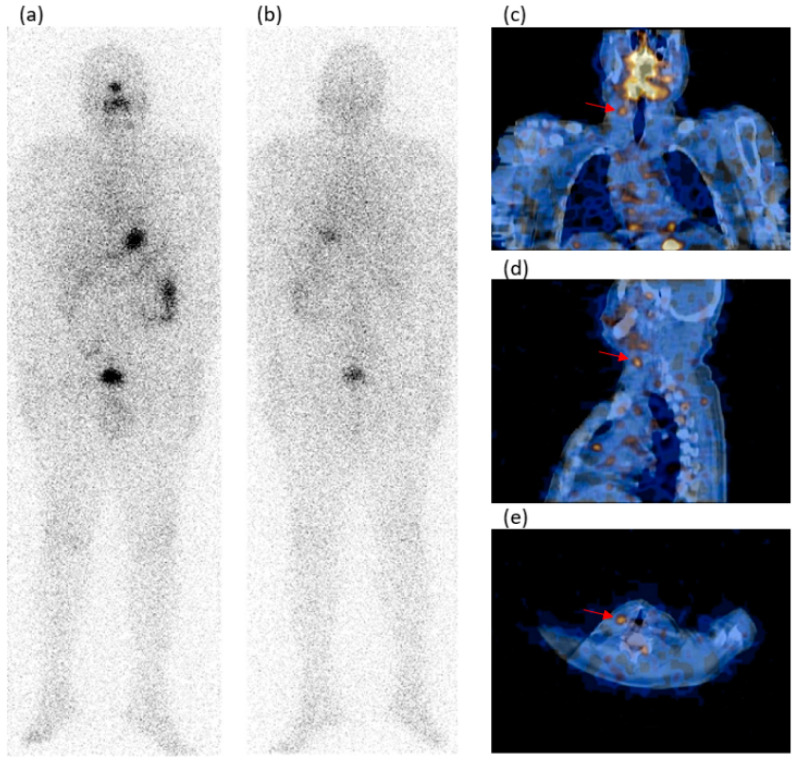
A 54-year-old male patient who underwent a total thyroidectomy and radioiodine ablation for a 30 mm T2 papillary carcinoma (PC) classic variant. During surgery, mETE and neck LN laterocervical metastasis were ascertained. During the follow-up, the WBS in anterior/posterior (**a**,**b**) views did not evidence pathologic radioiodine uptake foci. The SPECT/CT on the coronal/sagittal/transaxial (**c**–**e**) slides showed a radioiodine avid focus (red arrows) in the neck (right laterocervical region). This focus was classified as a metastatic lymph node and was submitted to a second surgery and radioiodine ablation. Thyroglobulin levels were in the hypothyroidism range before the present scintigraphy: 22 ng/mL; AbTg: absent.

In Figure 1 and Figure 2, there are two cases with neck LN metastasis, one T1b and one T2, with a positive SPECT/CT and negative WBS.

As reported previously, all 363 patients underwent a first radioiodine ablation after their total thyroidectomy. Afterwards, a second ablation was performed in all 61 patients who experienced metastases during the follow-up, either in the cases submitted for a second surgery or those in whom neoplastic lesions were difficult to achieve with surgery. Of these 61 patients, 18 underwent a further third dose of radioiodine; 7/18 had the appearance of new lymph node metastases only in the neck, and were not candidates for additional surgery, which was also the patients’ decision. The other 11/18 had persistent distant metastases (lung, mediastinum, and bone) with a partial response after the second radioiodine dose. Globally, in the patients with metastases during the follow-up, there were 79 radioiodine doses.

A total of 11/61 (18%) patients (5 T1b, 6 T2) had no risk factors during the primary tumor surgery, whereas in 50/61 (82%) metastatic patients (19 T1b, 31 T2), tissue risk factors were identified, such as mETE, neck LN metastases, and M/M. The percentage of patients with an initial low risk who developed metastases during the follow-up was 7.4%, and that of patients with an intermediate risk was 32.6% (*p* < 0.0001). Among the 50 patients with risk factors, mETE was present alone in 12 cases and associated with others in 22 cases, neck LN metastases were present alone in 8 cases and associated with others in 19 cases, and M/M was present alone in 6 cases and associated with others in 13 cases. In the patients with mETE alone during the surgery for the primary tumor, the neoplastic lesions during the follow-up were located largely in the neck: seven LTC, four PT, three SM, and two in distant metastases (one rib and one mediastinum). In the patients with neck LN metastases alone, the lesions were similarly located and largely in the neck: five LTC, two PT, four SC, while two metastases were in the lung, one in the mediastinum, one in the spine, and one in the rib. In the patients with M/M alone, all the lesions were present in the neck (eight LTC and five PT), except one in the lung.

As illustrated in Table 3, a univariate Cox regression analysis was performed to screen for significant variables associated with metastases during the follow-up in all the patients, taking into consideration both the T1b and T2 cases globally and individually. Considering the above risk factors, as well as the sex, age, and tumor size, the development of metastases during follow-up was significantly influenced by being male (only in T2 cases), mETE, and neck LN metastases of T2 size; M/M and age were not significant independent predictors of metastases.

A multivariate analysis, performed for all the patients, confirmed the significance of the above risk factors, as shown in Table 4.

By analyzing the patients with LN neck metastases and categorizing them according to their metastasis origin, the central compartment (N1a) and/or laterocervical (N1b), the Cox regression analysis revealed the presence of both N1a (HR: 3.17 [95%CI 1.12–8.94]) and N1b (HR: 4.37 [95%CI 2.21–8.65]) to be significant predictors of the disease’s adverse progression. However, the presence of N1b was more significant (*p* < 0.001 vs. *p* = 0.029), and the significance was even higher in patients with both N1a and N1b (HR: 6.40 [95%CI 3.23–12.68]; *p* < 0.0001). Thus, individuals with lateral lymph node involvement had a higher risk of recurrence compared to those with central lymph node involvement, and having both central and lateral involvement was associated with the highest risk.

Regarding the number of neoplastic lymph nodes, a univariate Cox model analysis showed a statistical significance (HR: 1.14 [95%CI 1.09–1.19]; *p* < 0.001), indicating an association between the number of lymph nodes and the risk of recurrence.

**Figure 3:**
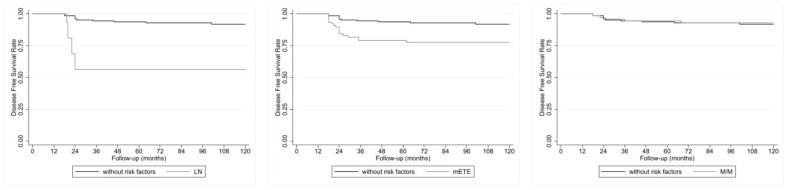
(**Left**) DFS in 16 patients with PC and LN metastasis alone and 154 without risk factors. Log-rank test: *p* < 0.001. (**Center**) DFS in 58 patients with PC and mETE alone and 154 matched patients with PC without risk factors. Log-rank test: *p* = 0.001. (**Right**) DFS in 74 patients with PC and M/M alone and 154 without risk factors. Log-rank test: *p* = 0.921.

As illustrated in Figure 3, after 120 months, the disease-free survival (DFS) of the metastatic T1b-T2 patients globally considered, with and without risk factors, appears significantly reduced for the log-rank test in the cases with neck LN metastases (56.3% vs. 91.9%; *p* < 0.001) and mETE (79% vs. 91.9%; *p* = 0.001), but not M/M (93% vs. 91.9%; *p* = 0.921).

**Figure 4:**
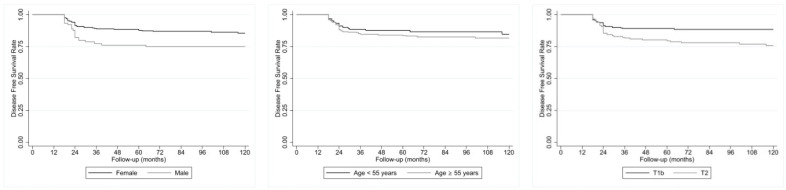
(**Left**) DFS in male (n. 90) and female (n. 273) patients, which is significantly (*p* = 0.007) reduced for male patients with log-rank test. (**Center**) DFS in patients with age of <55 (n. 239) and ≥55 (n. 124) years; the difference is not statistically significant with log-rank test (*p* = 0.482). (**Right**) DFS in both T1b patients with PC (n. 207) and T2 patients with PC (n. 156), which is significantly (*p* = 0.003) reduced for T2 cases with log-rank test.

As illustrated in Figure 4, comparing the DFS between male patients and female patients, this parameter is significantly shorter for male patients compared to female patients (75% vs. 85.7%; *p* = 0.007); no statistical difference in the DFS is present between patients with age <55 and ≥55 years (82% vs. 85%; *p* = 0.482). Furthermore, the DFS between the T1b and T2 patients shows a reduction in the T2 patients compared to the T1b patients (75.7% vs. 88.6%; *p* = 0.003), as illustrated in Figure 4.

Considering the T1b and T2 subgroup patients, mETE and LN metastasis caused a shorter DFS for patients without these risk factors. The T1b and T2 patients with LN metastases had a significantly shorter DFS when compared to patients without these risk factors (67.9% vs. 94.2%; *p* < 0.001 and 45% vs. 88.3%; *p* < 0.001, respectively). In addition, the T1b and T2 patients with mETE had a significantly shorter DFS when compared to patients without mETE (72.7% vs. 94.2%; *p* < 0.001 and 63.4% vs. 88.3%; *p* = 0.001, respectively).

Moreover, the metastases appeared in the majority of patients in the first 5 years of follow-up, while in some cases, they occurred long after period the thyroidectomy (>8 years), mainly in the T2 patients rather than the T1b cases (four cases vs. one).

The presence of risk factors did not affect mortality in this series of patients, as no patients died because of PC, and they are still alive and remain under observation.

## 4. Discussion

In the present study, metastases were ascertained during the follow-up in 16.8% of the patients (24 T1b and 37 T2), 18% of whom were without risk factors at the primary tumor surgery. In the remaining 82% of cases, at least one of the tissue risk factors (neck LN metastasis, mETE, and M/M) was present, with an associated increased risk of metastasis appearance in comparison with the patients without risk factors. The tumor size, patient age, and gender were also evaluated.

Regarding tumor size, in the different studies reported in the literature, it seemed to represent an important risk factor for neck LN metastasis appearance, particularly when it is >1 cm [13,26]. Based on some results [27], tumor size was even considered the greatest predictive value for lymph node recurrence, mainly when the tumor diameter is >2 cm, and emerged as an independent predictor of distant recurrence. In agreement with these last data, in the present study, the tumor size was a significant risk factor for metastasis appearance, as confirmed by a Cox regression multivariate analysis, especially when the dimension was >2 cm, notwithstanding that approximately 40% of the patients with metastases were classified as T1b. In addition, the DFS results were shorter for the T2 patients during the follow-up, compared with the T1b cases. However, as has been reported in the literature, even a size ≤1 cm, as in microcarcinoma, can be associated with regional metastasis appearance, although with less risk [28,29,30].

In the present series of patients, no statistical significance for the Cox regression univariate analysis was observed regarding age as an independent risk factor for metastasis appearance and DFS reduction, since no difference was present between the patients <55 and ≥55 years, as also observed by other authors [31]. However, in other studies (printed before the *AJCC* eighth edition publication, which increased the age cutoff for risk stratification in papillary thyroid carcinoma from 45 to 55 years), an age ≥45 years was reported as associated with an unfavorable prognosis in patients with PC, and the carcinoma death rate was observed to increase with older age [32]. Nevertheless, an age <45 years resulted in a poor predictor of LN appearance, as reported by other authors [33,34]. In a further study reported in the literature, PC progression was less favorable in patients with an age under 20 years or older than 60 years [35].

In addition, in the present study, although the number of patients who experienced metastases was higher in females, only the male gender proved a significant risk factor for metastases appearance; however, this significance only resulted for the T2 patients from the Cox regression univariate and multivariate analyses. The role of the male gender in the progression of the disease is in agreement with the results obtained by other authors, who have reported that the male gender is a significant risk factor for LN appearance in patients with PC during the follow-up, and this factor was associated with some unfavorable clinical–pathological features and with more advanced disease [13,36,37,38]. However, the role of the male gender is still controversial since, in other studies, this factor was not associated with a recurrence risk [39], and did not seem to be an independent prognostic factor for disease-specific survival [40].

Furthermore, in the patients of the present study, M/M did not turn out to be a significant predictor of metastasis appearance during the follow-up. Moreover, M/M showed no impact on DFS, in agreement with the results of other authors who have rejected the unfavorable role of M/M on patient clinical outcomes and an association with distant metastasis appearance and mortality [41]. However, in other studies, M/M has been labeled as a higher risk factor for neck LN metastasis appearance during the follow-up, compared with tumor single focus [13,42], and even more so when the tumor size is >1 cm [42,43].

In the present study, the development of metastases during the follow-up and the reduction in DFS were significantly influenced, among the other risk factors, by the presence of neck LN metastases during the surgery for the primary tumor, mostly originating in the laterocervical regions. The presence of both N1a and N1b neoplastic lymph nodes were a significant predictor of unfavorable tumor prognosis, but the N1b cases showed a higher significance compared to the N1a, while both of these had less significance than the cases with neoplastic lymph nodes in the two compartments. It is well known that the presence of neck LN metastasis in patients with PC at the initial surgery represents a sign of the aggressive progression of the disease during the follow-up, as also reported in the *AJCC* eighth edition. Neck LN metastases are considered the most common high-risk factors and independent predictors for the appearance of recurrences and the reduction in DFS during the disease course, in particular in older patients [18,44,45], and even more when other characteristics of the LN are evaluated, such as LN size and number. In this regard, an increase in the dimension and number of metastatic LNs at tumor diagnosis may contribute more to predicting loco-regional and distant metastases during the follow-up. This increase significantly affects the DFS [15,23,26], especially when including extra-nodal extension [46]. Consequently, a more aggressive treatment for carcinomas in this condition is recommended [19]. Also in the present study, the number of neoplastic LNs was evaluated, and it emerged as a significant risk factor for metastasis occurrence from the Cox regression univariate analysis.

Regarding mETE, the team of pathologists who gave their opinion on the histopathological specimens from the patients in this study, in order to classify the cases with this risk factor, as follows below, adopted the classical definition of mETE: an extra thyroid tumor extension beyond the thyroid capsule with the invasion of the perithyroid soft tissue and sternothyroid muscle microscopically evidenced. The pathologist team has modified the diagnostic criteria for mETE over the years, based on international pathology guidelines and discussions within the Multidisciplinary Group involved in thyroid cancer diagnosis, and the team, in this sense, has reviewed the histological specimens analyzed in past years.

The data from this retrospective study show that this factor significantly influenced the disease progression, with unfavorable outcomes for the patients, as confirmed by the univariate and multivariate Cox regression analyses, especially when the carcinomas were >2 cm; the DFS reduction was evidenced by a log-rank test. During the follow-up period, 20.7% of the patients who had mETE alone developed metastases, whereas only 7.1% of the patients who did not have tissue risk factors presented with metastatic lesions (*p* = 0.005). Thus, mETE, also alone, could be a predictive risk factor for metastasis occurrence, potentially causing a worse disease prognosis when compared to patients without mETE. Conflicting results have been reported in numerous studies on the prognostic relevance of mETE. About that, some authors have identified a poor favorable prognosis in patients with PC and mETE when compared with those without mETE, considering this risk factor detrimental for the progression of the disease [26,47,48,49,50,51]. However, other authors have not considered mETE to be an independent predictor of the appearance of recurrences or metastases, and have not identified any impact on survival in patients with associated mETE [52,53,54,55,56,57,58]. These conflicting data prompted researchers to make changes to the international diagnostic strategies in the *American Joint Committee on Cancer (AJCC) Staging System* [25]. In particular, the hypothesis that mETE does not interfere with the clinical outcome of the affected patients prevailed. Thus, in agreement with this hypothesis, the standards reported in the seventh edition were revised in the *AJCC* eighth edition, removing mETE from the Staging System due to its negligible effect on tumor prognosis. However, the problem is still strongly debated and the decision to remove mETE from the *AJCC* eighth edition should be re-considered based on other results obtained from a larger number of cases, including those obtained in the present study, where PC in the patients with mETE resulted in more aggressive disease than in the cases without mETE. This risk factor, even alone, could be considered the cause of an unfavorable disease prognosis in those patients in whom the other risk factors are absent. The *AJCC* eighth edition’s omission of mETE could compromise patient care and management; thus, it is recommended that mETE be added to the risk stratification model in a new *AJCC* edition.

However, interpreting mETE results from different pathologists is a complex process that depends on many morphologic features, resulting in controversial results [59]. In this regard, this explains why the definition of mETE has undergone some changes in the last few years. However, not all pathologists have accepted these changes, and there is disagreement about the definition of mETE and discrepancies in the criteria for its identification. Moreover, the pathologic diagnosis of mETE is not readily reproducible, which suggests the necessity of standardizing the histopathologic criteria for mETE.

Furthermore, no relationship with mortality was found for either the patients with or without risk factors, since during this study no patient died from thyroid carcinomas. In addition, in no cases were thyroid immune diseases ascertained, such as Graves’ and Hashimoto’s diseases, that, in some studies, have been reported as having an unfavorable impact on PC recurrence [60,61,62], even if these data were not confirmed by others [13,63,64,65].

The identification of metastases during follow-up was achieved by traditional and advanced diagnostic imaging procedures. The ^131^I SPECT/CT has always been performed on all patients, among other diagnostic procedures, and it has been proven to be effective in identifying metastases in patients with PC, both with and without risk factors. The performance of the SPECT/CT was significantly better than that of the traditional WBS, due to the tomographic procedure revealing a significant increase in metastatic lesions. The reliable results reported in the literature [66,67,68,69,70] are confirmed by the data obtained in the present study. Thus, SPECT/CT is recommended for routine use in the PC diagnostic protocol during the follow-up after a thyroidectomy and radioiodine ablation.

Regarding the results obtained in the present study, one future development could be the use of artificial intelligence (AI) methods. AI could be able to analyze the results obtained using neural networks more effectively, which could assist imaging procedures in predicting the appearance of metastases and the progression of the disease, as has been suggested by some studies in the literature [71,72]. In this way, both the efficiency and effectiveness of diagnostic procedures could be increased, with a more correct diagnosis of metastases and the management of affected patients.

Some limitations of this study must be acknowledged. First, it is important to note that this study is retrospective, and some information on patient data may have been lost or not reported in the medical records. Moreover, this study only involves one center and thus is limited to a small number of patients, which, however, could also be due to the very narrow exclusion criteria. The present study has multiple strengths, including similar criteria within the same department for both post-surgical therapy and the diagnostic strategy used during follow-up. However, a single-center study makes the reproducibility of the exams more difficult, particularly regarding the imaging procedures, with results that may not be generalizable. In addition, this study fails to provide information on genetic alterations that could impact patient outcomes. Second, although the average follow-up period is long, it may not be long enough for the identification of recurrences and/or metastases by clinical and imaging examination; in addition, missed neoplastic lesions may occur, considering the fact that they can also appear later. Third, the absence of histological results for certain metastases is a limitation because of the inability to find their sites or the inability to perform invasive interventions, which also has practical and ethical implications. Determining the nature of metastases in these patients is only possible through a long-term follow-up that involves sequential clinical and imaging procedures, as well as evaluations of serum thyroglobulin level changes.

## 5. Conclusions

T1b-T2 patients with PC and neck LN metastases and mETE during the initial surgery, particularly males, showed a significant increase in disease progression and shorter DFS. Despite the widespread awareness of LN metastasis as a risk factor for developing metastasis during follow-up, the role of mETE is currently being debated, as some studies have reported it to have little impact on tumor prognosis. However, according to the findings of this study, this risk factor, even alone, seems to be a significant predictor of negative disease progression. The classification of patients with T1b-T2 PC with mETE in the next *AJCC* might be revised if more cases confirm the data of the current study and those with comparable results. It is suggested to monitor T1b-T2 patients with PC and mETE closely.

## Figures and Tables

**Table 1:** Demographics, tumor sizes, nodule or multinodular goiter conditions, ATA risk stratification, and TNM classification (*AJCC* eighth edition) were analyzed in 363 patients with PC during surgery on primary tumor.

Characteristics	All 363 Patients	T1b 207 Patients	T2 156 Patients
Age (years), median (IQR)	49 (39–59)	49 (40–59)	49 (36.5–57)
Age, <55/≥55 years	239/124	131/76	108/48
Sex, females/males (F/M)	273/90	162/45	111/45
Tumor size (mm), median (IQR)	17 (13–25)	14 (11–15)	25 (22.5–30)
Thyroid nodules	282	156	126
Multinodular goiter	81	51	30
ATA risk stratification			
Intermediate (I)	135	62	73
Low (L)	228	145	83
TNM (*AJCC* eighth)			
T1b N0M0	179	179	
T1b N1M0	28	28	
T2 N0M0	126		126
T2 N1M0	30		30

**Table 2:** One or more risk factors were ascertained at the time of surgery on the primary tumor in 209/363 patients with PC.

Risk Factors	All Cases	T1b	T2
mETE alone	58	29	29
Neck LN metastasis alone	16	8	8
(M/M) alone	74	52	22
mETE + LN	18	7	11
mETE + M/M	19	5	14
LN + M/M	13	6	7
mETE + LN + MM	11	7	4

mETE: minimal extrathyroid tumor extension; LN: lymph node; (M/M): multifocality/multicentricity.

**Table 3:** Cox regression univariate analysis to evaluate relationship between demographic and histologic characteristics and metastasis development during 120 months of follow-up.

Risk Factors	All Patients	T1b Patients	T2 Patients
Male sex	2.01 [95%CI 1.19–3.38] *p* = 0.009	0.98 [95%CI 0.36–2.63] *p* = 0.963	2.79 [95%CI 1.46–5.33] *p* = 0.002
mETE	3.57 [95%CI 2.14–5.96] *p* < 0.001	4.85 [95%CI 2.12–11.06] *p* < 0.001	2.50 [95%CI 1.30–4.80] *p* = 0.006
LN metastases	4.73 [95%CI 2.83–7.90] *p* < 0.001	4.87 [95%CI 2.10–11.28] *p* < 0.001	4.27 [95%CI 2.23–8.17] *p* < 0.001
T2 size	2.18 [95%CI 1.30–3.68] *p* = 0.003		
M/M	0.86 [95%CI 0.49–1.49] *p* = 0.583	0.52 [95%CI 0.19–1.41] *p* = 0.201	1.20 [95%CI 0.61–2.37] *p* = 0.590
Age	1.21 [95%CI 0.70–2.11] *p* = 0.487	1.72 [95%CI 0.68–4.36] *p* = 0.254	0.87 [95%CI 0.44–1.74] *p* = 0.699

**Table 4:** Cox regression multivariate analysis to evaluate relationship between demographic and histologic characteristics and metastasis development during 120 months of follow-up.

Risk Factors	All Patients	T1b Patients	T2 Patients
Male sex	1.75 [95%CI 1.04–2.95] *p* = 0.037	0.96 [95%CI 0.35–2.62] *p* = 0.929	2.64 [95%CI 1.37–5.10] *p* = 0.004
mETE	2.59 [95%CI 1.53–4.38] *p* < 0.001	3.59 [95%CI 1.49–8.67] *p* = 0.004	2.05 [95%CI 1.05–3.99] *p* = 0.035
LN metastases	3.70 [95%CI 2.20–6.22] *p* < 0.001	3.20 [95%CI 1.30–7.87] *p* = 0.011	4.32 [95%CI 2.24–8.31] *p* < 0.001
T2 size	1.73 [95%CI 1.02–2.93] *p* = 0.042		

## Data Availability

The data that have been presented in this study are available on reasonable request from the corresponding author.

## Abbreviations

*AJCC*	American Joint Committee on Cancer
ATA Management Guidelines	American Thyroid Association Management Guidelines
PC	Papillary Thyroid Carcinoma
ETE	Extrathyroid Tumor Extension
eETE	Extended Extrathyroid Tumor Extension
mETE	Minimal Extrathyroid Tumor Extension
LN Metastasis	Lymph Node Metastasis
LTC	Cervical Lateral Lymph Node Metastasis
SM	Sub/Retromandibular Lymph Node Metastasis
PT	Paratracheal Lymph Node Metastases
SC	Supraclavicular Lymph Node Metastasis
M/M	Multifocality/Multicentricity
DFS	Disease-Free Survival
TSH	Thyroid Stimulating Hormone
rh-TSH	Recombinant Human TSH
AbTg	Thyroglobulin Antibody
AbTPO	Thyroid Peroxidase Antibody
TRAbs	TSH Receptor Antibody
US	Ultrasound
FNAB	Fine-Needle Aspiration Biopsy
^131^I WBS	^131^I Whole-Body Scan
^131^I SPECT/CT	^131^I Single-Photon Emission Computerized Tomography/Computerized Tomography
^18^F-FDG PET/CT	^18^F Fluorodeoxyglucose Positron Emission Tomography/Computerized Tomography
CT	Computerized Tomography
MRI	Magnetic Resonance Imaging
AI	Artificial Intelligence
